# An investigation of drug-resistant *Acinetobacter baumannii* infections in a comprehensive hospital of East China

**DOI:** 10.1186/s12941-015-0066-4

**Published:** 2015-02-03

**Authors:** Su-ying Zhao, Dong-yang Jiang, Peng-cheng Xu, Yi-kai Zhang, Heng-fang Shi, Hui-ling Cao, Qian Wu

**Affiliations:** Department of Laboratory Medicine, Jiangsu provincial hospital of Traditional Chinese Medicine, Nanjing, China; The State Key Laboratory of Reproductive Medicine, Nanjing Medical University, Nanjing, China; Department of Hygienic Analysis and Detection, School of Public Health, Nanjing Medical University, Nanjing, 211166 China; Department of Environmental Health Sciences, Bloomberg School of Public Health, Johns Hopkins University, Baltimore, USA

**Keywords:** *Acinetobacter baumannii*, Drug-resistant gene, PCR, PFGE

## Abstract

**Background:**

To investigate the drug resistant gene profiles and molecular typing of *Acinetobacter baumannii* isolates collected from clinical specimens in a comprehensive hospital, Jiangsu province.

**Methods:**

This study included 120 patients in a comprehensive hospital with drug-resistant *A. baumannii* infections on clinical specimens from October 2011 to December 2013. Antibiotic susceptibility test was determined by Vitek 2 Compact system. *OXA*-51, *OXA*-23, *OXA*-24, *OXA*-58, *VIM*, *IMP*, *SHV*, *GES*, *TEM*, *AmpC*, *qacE*Δ1-*sul*1, *intI* l, *CarO*, *aac*(6′)-*Ib*, and *aac(6′)-II* were analyzed by PCR. The analysis of molecular typing for 50 multidrug resistant *A. baumannii* isolates was performed by PFGE.

**Results:**

A total of 64(53%) isolates were multidrug-resistant *A.baumannii*. The antibiotic susceptibility tests showed that the resistant rates to common antibiotics of mutidrug-resistant *A. baumannii* were extremely high, most of which over 60%. One hundred and ten isolates harbored *OXA-51* (91.7%), 100 for *OXA-23*(83.3%), 103 for *VIM-1*(85.8%), 90 for *AmpC*(75.00%), 50 for *aac(6′)-Ib*(41.7%), 77 for the loss of *CarO* (64.2%), 85 for *intl1*(70.8%), and 64 for *qacE*Δ*1-sul1*(53.33%), while *OXA-24* was undetected. Fifty multidrug-resistant *A. baumannii* isolates belong to 14 clones according to the PFGE DNA patterns. Main clone A includes 24 isolates, while clone B and clone C includes 6 and 9 isolates, respectively and others with no common source identified.

**Conclusion:**

There is high morbidity of *A. baumannii* infections in the hospital, especially in ICU and sputum is the most common sample type.The mainly drug-resistant genes of *A. baumannii* are *OXA-51*, *OXA-23*, and *VIM-1* in the hospital. Clonal dissemination provides evidence for the prevalence of multidrug-resistant *A. baumannii* among clinical isolates. It is suggested that there is an urgent need for effective control and prevention measures.

## Background

*Acinetobacter baumannii (A.baumannii)* is a non-fermentative, gram-negative, conditional pathogenic bacterium, which can colonize and survive for prolonged period under a wide range of environmental conditions, such as hospital environment and human skin [1]. Meanwhile, *A. baumannii* is an emerging opportunistic nosocomial pathogen, with increasingly global prevalence, responsible for a variety of nosocomial infections including nosocomial pneumonia, blood infection, urinary tract infection, surgical wound infection, etc., especially for patients in intensive care unit (ICU) [2,3]. Its great capacity to survive in low-moisture environments and its ability to develop resistance to antimicrobial agents afford *A. baumannii* the possibility of spreading in hospitals. The increasing threat of antibiotic resistance in microbes impacting on the patient outcomes has been recognized as a challenge for treatment of clinical infection with broad spectrum antibiotics use. According to the report from CHINET, 2012, surveillance data reveal that the resistance rates of Acinetobacter spp. (*A. baumannii* accounted for 89,6% to imipenem and meropenem were up to 62.8%and 59.4%, respectively [4]. What’s more, multidrug resistant *A. baumannii*, defined as resistant to at least three different groups of antibiotics, causes numerous nosocomial outbreaks and health care–associated infections around the world [1]. The identification of drug resistance mechanisms in *A. baumannii* will improve the outcome of infections caused by this organism. The resistance mechanisms of *A. baumannii* are complex, which include activating or production of enzyme, the integron formation, outer membrane permeability, biofilm formation, drug exocytosis mechanism and so on [5-9].

Carbapenem resistance in *A.baumannii* is increasingly being observed worldwide [5]. The most important mechanism of carbapenem resistance in *A. baumannii* is the enzymatic hydrolysis mediated by carbapenem-hydrolyzing β-lactamases, including class A *(*TEM, SHV, and GES), class B (IMP, VIM, and SIM), class C (AmpC), and class D (OXA-23-like, OXA-24-like, OXA-51-like, and OXA-58-like) [10]. Resistance to aminoglycosides in *A. baumannii* is mainly mediated by the production of aminoglycosides-modifying enzymes (AMEs). The most frequent AMEs in *A. baumannii* are AAC(6′)-Ib and AAC(6′)-II [3,11]. What’ more, the loss of outer-membrane protein(OMP) and the acquisition of class 1 integron are also contributed to an increasing incidence of drug resistance [12]. In addition, many commercial products based on ammonium quaternary compounds (QAC) are currently used in considerable quantities as antiseptic agents in hospitals, but due to the intrinsic and chronic resistance to QAC, infections with A. baumannii are growing [13].

To date, there are few reports of combinations of different resistance mechanisms, but there is a correlation of antimicrobial resistance with the enzyme production and porin and integrons. In the present study, from 2011 to 2013, surveillance at a provincial hospital in Jiangsu detected 120 drug-resistant *A. baumannii* isolates. We aimed to analyze the genetic linkage and drug-resistance gene profiles of these drug-resistant *A. baumannii* isolates and investigate various mechanisms of drug resistance in isolates.

## Materials and methods

### Subjects and bacterial isolates

This investigation was conducted at a comprehensive hospital in Nanjing, Jiangsu, China, from Oct 2011 to Dec 2013. Written informed consent was obtained from the participants for the use of samples in this study. This study was approved by the Nanjing Medical University Clinical Research Ethics Committee, Nanjing, China. No patients received antibiotic therapy before samples were collected. All the clinical isolates were routinely collected and stored at −80°C until use. One hundred and twenty *A. baumannii* strains were isolated from clinical samples. Multidrug resistant *A. baumannii* was defined as *A. baumannii* isolates which were resistant to more than 3 classes of antimicrobials.

### Antibiotic susceptibility testing

*A. baumannii* identification and general antimicrobial susceptibilities were performed using Vitek 2 Compact system (bioMérieux, Inc., Marcy-l’Etoile, France). *Escherichia coli* (ATCC 25922), *Pseudomonas aeruginosa* (ATCC 27853), and *Staphylococcus aureus* (ATCC 29213) were used as quality control strains. Susceptibility results were interpreted according to the Clinical and Laboratory Standards Institute (CLSI) guidelines.

### PCR of drug resistant genes

Genomic DNA of *A.baumannii* isolates was extracted using TIANamp Bacteria DNA Kit(Tiangen). PCR was performed using Taq PCR Master Mix(TaKaRa).Primers were synthesized by Jierui, Shanghai. The details of primer sequences were showed in Table 1. Each reaction was performed in a final volume of 50 μl consisting of 25 μl Taq Mix, 1 μl primers, 1 μl DNA template and 22 μl RNase Free H_2_O. OXA-51 and 16S rRNA was used as the internal control. The cycling conditions were as follows: an initial denaturation step at 94°C for 5 min, followed by 30 cycles at 94°C for 30 s, 55°C for 30 s and 72°C for 90 s. Then the PCR products were electrophoresed in agarose gel to detect the target band.

**Table 1:** **The sequence of primers used in this study**

**Target gene**	**Primer**	**Sequence**	**product size(bp)**
bla_OXA_-51-like	OXA-51-like F	TAATGCTTTGATCGGCCTTG	353
	OXA-51-like R	TGGATTGCACTTCATCTTGG	
bla_OXA_-23-like	OXA-23-like F	GATCGGATTGGAGAACCAGA	501
	OXA-23-like R	ATTTCTGACCGCATTTCCAT	
bla_OXA_-24-like	OXA-24-like F	TTCCCCTAACATGAATTTGT	1024
	OXA-24-like R	GTACTAATCAAAGTTGTGAA	
bla_OXA_-58-like	OXA-58-like F	TGGCACGCATTTAGACCG	507
	OXA-58-like R	AAACCCACATACCAACCC	
bla_IMP-1_	IMP F	CTACCGCAGCAGAGTCTTTAC	587
	IMP R	AACCAGTTTTGCCTTACCAT	
bla_VIM-1_	VIM F	ATTCCGGTCGGMGAGGTCCG	633
	VIM R	GAGCAAGTCTAGACCGCCCG	
bla_SHV_	SHV F	GGTTATGCGTTATATTCGCC	865
	SHV R	TTAGCGTTGCCAGTGCTC	
bla_GES_	GES F	ATGCGCTTCATTCACGCAC	392
	GES R	ATTTGCTGATTTCGCTCGG	
bla_TEM_	TEM F	ATCAGCAATAAACCAGC	516
	TEM R	CCCCGAAGAACGTTTTC	
16S rRNA	16S-8F	AGAGTTTGATCCTGGCTCAG	1499
	16S-1493R	ACGGCTACCTTGTTACGACTT	
bla_AmpC_	AmpC F	CGACAGCAGGTGGAT	513
	AmpC R	GGTTAAGGTTGGGATG	
aac(6′)-Ib	aac(6′)-Ib F	ATGACTGAGCATGACCTTGC	519
	aac(6′)-Ib R	TTAGGCATCACTGCGTGTTC	
aac(6′)-II	aac(6′)-II F	GAGCGACCGACTCTTGATG	326
	aac(6′)-II R	CGTATGGCTCGATGGTTGTT	
CarO	CarO F	CAGAGCCTTTTCCTAAGGAGAA	916
	CarO R	GCTCACCTGATGCTGACATTAA	
qacΔE 1-sul1	qacEΔ1-sul1 F	TAGCGAGGGCTTTACCTAAGC	300
	qacEΔ1-sul1 R	ATTCAGAATGCCGAACACCG	
intl 1	intl 1 F	ACGAGCGCAAGGTTTGGT	565
	intl 1 R	GAAAGGTCTGGTCATACATG	

### Molecular typing *by pulsed-field gel electrophoresis*

Pulsed-field gel electrophoresis (PFGE) was performed using previously described methods [14]. In brief, the purified bacterial genomic DNA was digested by the restriction enzyme ApaI( TaKaRa), and the fragments were separated in a CHEF Mapper system (Bio-Rad Laboratories, Hercules, CA, USA) with pulses ranging from 5 to 20 seconds at a voltage of 5 V/cm and switch angle of 120° for 19 hours at 14°C. And then gels were stained with ethidium bromide and DNA patterns were acquired by Bio-Rad Vilber Lourmat. The PFGE profiles were interpreted according to Tenover *et al.* [15]. We used BioNumerics software (Applied Maths, Kortrijk, Belgium) to analyze similarities between digitized PFGE outputs. The Between-groups linkage method was used to analyze hierarchic clustering. The classification criteria is as followed:(1) The completely consistent patterns are defined as the same clone, for example, Clone A. (2) If there are 1 to 3 different band compared with Clone A, define it as subtype (Clone A_1_…Clone A_n_). (3)If there are over 3 different stripes, define it as another clone, for example, Clone B, Clone C, and Clone D etc.

### Statistical analysis

Statistical analyses were performed using SPSS18.0. P values <0.05 are considered statistically significant. Statistical significance was assessed via χ^2^ test of Fisher’s exact test for categorial variables and Student’s test or the Mann–Whitney U test for continuous variables.

## Results

### The characteristics of drug-resistant A. baumannii infection subjects

During the study period there were 120 strains isolated from adults aged 23 to 98 years old. The average age of the patients was 73.43 ± 13.09 years, and male–female ratio was 1.24:1. The age distribution were shown as followed in Table 2. *A. baumannii* isolates were mainly distributed in respiratory department, ICU, and emergency department, accounting for 21.7%, 16.7% and 11.7%, respectively. while multidrug-resistant strains, were mainly in ICU and emergency department, accounting for 29.7% and 20.3%, respextively. According to Chi-square test, there were statistical differences in the incidence of multidrug-resistant strains among different wards (χ2 = 195.504, P < 0.001) (Table 3). The strains were mainly isolated from sputum(112, 93.3%), and the others were from blood, urine, endotracheal tube suction, bronchoalveolar and so on.

**Table 2:** **Age distribution between patients with multidrug-resistant and non-multidrug-resistant** ***A. baumannii***

**Age**	**No. of patients with multidrug-resistant**	**No. of patients with non-multidrug-resistant**	**Total number**
<40	2	0	2
40~	2	2	4
50~	1	6	7
60~	13	9	22
70~	25	21	46
>80	21	18	39
Total	64	56	120

**Table 3:** **Ward distribution between patients with multidrug-resistant and non-multidrug-resistant** ***A. baumannii***

**Ward**	**No. of patients with multidrug-resistant**	**No. of patients with non-multidrug-resistant**	**Total number**
Respiration	6	20	26
ICU	19	1	20
Emergency	13	1	14
Geriatrics	5	8	13
Nephrology	2	6	8
Neurology/neurosurgery	12	2	14
Cardiology	4	4	8
Others*	3	14	17

*Others: Department of wounds, rheumatology, Chinese Acupuncture, E.N.T., oncology, gastroenterology, hematology and cardiothoracic surgery.

### Antimicrobial susceptibility of A. baumannii isolates

The susceptibility patterns of the 120 *A. baumannii* strain were shown in Table 4. Among 120 drug resistant strains, 64 were multidrug resistant. Most exhibited resistance across different classes of agents notably imipenem, gentamicin, ampicillin/sulbactam, ceftazidime, and ciprofloxacin.

**Table 4:** **Antibiotics resistance of multidrug-resistant** ***A. baumannii*** **isolates**

**Antibiotics**	**Multidrug-resistant group (n = 64)**	
	**number**	**rate (%)**
Ampicillin	51	78.5
Cefotetan	50	76.9
Cefazolin	51	78.5
Ceftriaxone	49	75.4
Imipenem	60	92.3
Gentamicin	57	87.7
Levofloxacin	52	80.0
Furadantin	51	78.5
Ampicillin/sulbactam	61	93.8
Piperacillin/tazobactam	58	89.2
Ceftazidime	60	92.3
Cefepime	60	92.3
Aztreonam	60	92.3
Ciprofloxacin	64	98.5
Cotrimoxazole	53	81.5
Tobramycin	53	81.5
Cefoperazone	14	21.5
Cefoperazone/sulbactam	12	18.5

### The detection of drug-resistant genes in these A. baumannii isolates

All 120 isolates were identified as *A. baumannii* using 16S rRNA gene validation. With regard to the 120 isolates, one hundred and ten strains carried *OXA-51* gene characteristic of *A. baumannii*. The *OXA-23* genes were carried by 100 strains. Coexistence of the *OXA-51* and the *OXA-23* genes was detected in 95 strains. Only one strain harbored *OXA-58* gene, and all strains were negative for *OXA-24* gene. In multidrug resistant strains, 73.4% of which were positive for *SHV* gene, as Amber class A of β-lactamases; 95.3% for *VIM*, one of MBL genes; 82.8% for *AmpC* β-lactamases; 50.0% for *aac(6′)-Ib*, the most frequent aminoglycoside-modifying enzymes; 32.8% for *CarO*, outer membrane protein; 82.8% for class 1 integrase( *Intl 1*); 56.3% for *qac△E1-sul1*, which is related to resistance to quaternary ammonium compounds and sulfonamide (Table 5).

**Table 5:** **The detective rate of drug-resistant genes between groups**

**Gene**	**Total rate (%)**	**Multidrug-resistant group (n = 64)**		**Non-multidrug-resistant group (n = 55)**	
		**No.**	**rate (%)**	**No.**	**rate (%)**
bla_OXA_-51-like	91.7	64	100.0	46	82.1
bla_OXA_-23-like	83.3	58	90.6	42	75.0
bla_OXA_-24-like	0	0	0	0	0
bla_OXA_-58-like	0.8	1	1.5	0	0
bla_IMP-1_	5.0	2	3.1	4	7.1
bla_VIM-1_	85.8	61	95.3	42	75
bla_TEM_	25.8	16	25.0	15	26.8
bla_SHV_	66.7	47	73.4	33	58.9
bla_GES_	3.3	1	1.6	3	5.4
16S rRNA	100.0	65	100.0	55	100.0
bla_AmpC_	75.0	53	82.8	37	66.1
aac(6′)-Ib	41.7	32	50.0	18	32.1
aac(6′)-II	2.5	1	1.6	2	3.6
Loss of CarO	64.2	43	67.2	34	60.7
Intl 1	70.8	53	82.8	32	57.1
qac∆E1-sul1	53.3	36	56.3	28	50.0

### PFGE typing of multidrug-resistant A. baumannii isolates

**Figure 1:**
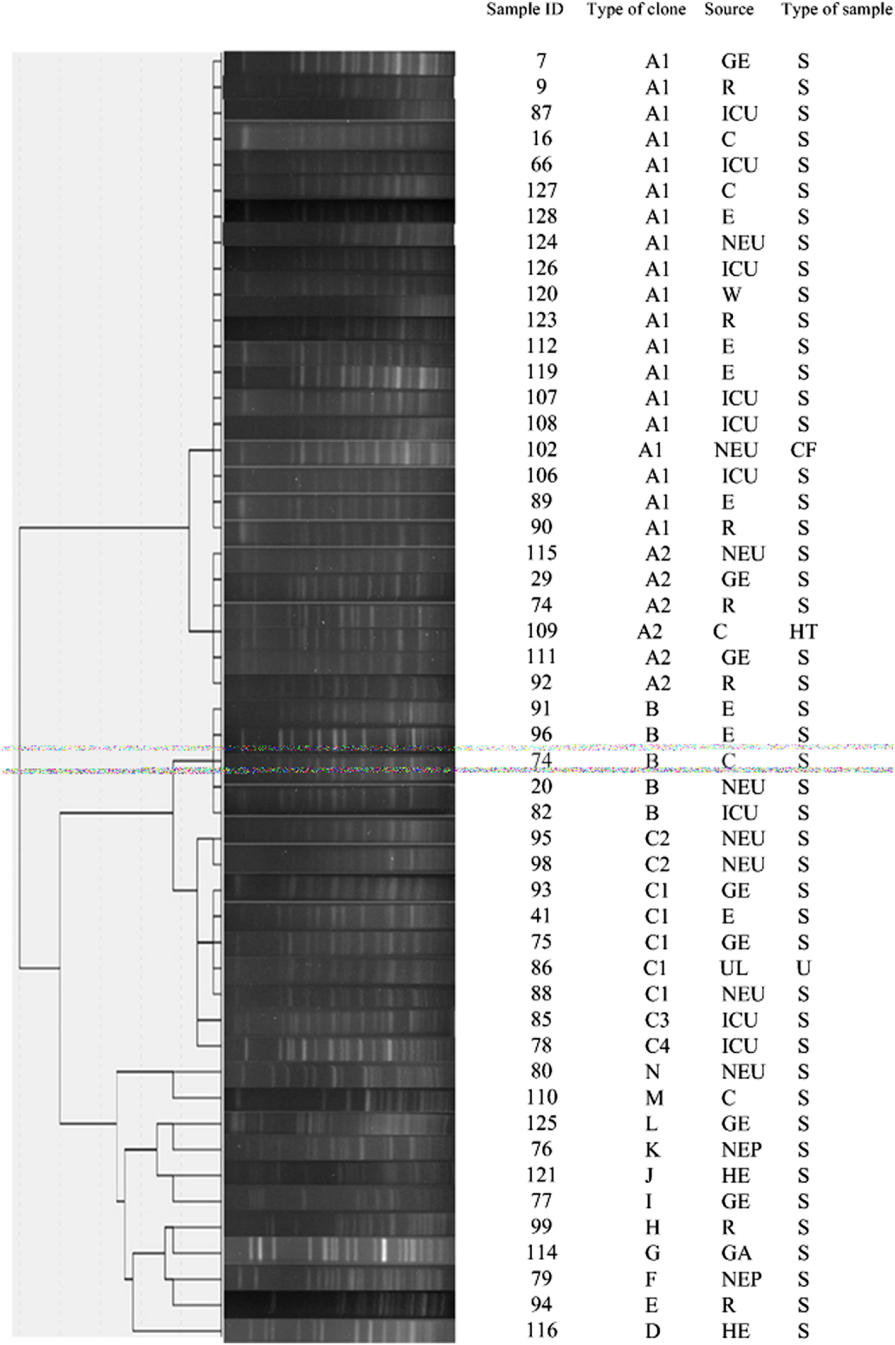
**Molecular typing of representative multidrug resistant** ***A. baumannii*** **isolates by PFGE.** Participating wards, clone type, and source of the samples were also indicated. GE: Geriatrics; R: Respiration; C: Cardiology; E: Emergency; NEU: Neurology; W:wound; GA: Gastroenterol; NEP: Nephrology; HE: Hematology; UL: Urology; S:sputum; CF: cerebrospinal fluid; U:urine.

Figure 1 showed that the PFGE results of the multidrug resistant *A. baumannii* isolates. More than 25 DNA fragments were observed in each isolate. The results of genetic linkage were presented as a dendrogram. PFGE revealed that the isolates had diversity with multivariate clones. Fifty strains were typed into 14 clones. Clone A had 25 isolates (50%), including A_1_ for 19 , and A_2_ for 6. Clone B had 5. Clone C had 9,including C_1_ for 5, C_2_ for 2, and C_3_ and C_4_ for 1,respectively. The others had no common resource. Clonal distribution was shown as followed in Table 6.

**Table 6:** **The distribution of clone strains in each ward**

**Ward**	**Clone**																	
	**A1**	**A2**	**B**	**C1**	**C2**	**C3**	**C4**	**D**	**E**	**F**	**G**	**H**	**I**	**J**	**K**	**L**	**M**	**N**
ICU	6	0	1	0	0	1	1	0	0	0	0	0	0	0	0	0	0	0
Respiration	3	2	0	0	0	0	0	0	0	0	0	0	0	1	0	1	0	0
Neurology	2	1	1	1	2	0	0	0	0	0	1	0	0	0	0	0	0	0
Emergency	4	0	2	1	0	0	0	0	0	0	0	0	0	0	0	0	0	0
Geriatrics	1	2	0	2	0	0	0	1	0	1	0	0	0	0	0	0	0	0
Others*	3	1	1	1	0	0	0	0	1	0	0	1	1	0	1	0	1	1

*Others refer to Department of Cardiology, Urology, Nephrology, Hematology, and Gastroenterology.

## Discussion

*Acinetobacter baumannii*, recently as an increasingly common pathogen, is closely associated with hospital acquired infection [16]. *A. baumannii* has the characteristics of strong viability and rapid development of drug resistance ability and has raised an important challenge to our therapeutic approach [17]. In this paper, we characterized the occurrence of drug resistant of *A. baumannii* isolates in a hospital in Nanjing. From 2011 to 2013, a total of 120 drug-resistant isolates were collected. The source of 120 strains was mainly from older patients. There were 107 patients aged more than 60 years old (89.2%). The main ward for drug resistant *A. baumannii* isolate is ICU and respiratory department. The main source of *A. baumannii* isolates was respiratory specimen. These were consistent with data presented by others [18]. In other words, respiratory tract infection is the common manifestation of drug resistant *A. baumannii* infection.

Overall, the resistance rates were high for most antimicrobial agents. The resistance rate to imipenem, ampicillin/sulbactam, ceftazidime, cefepime, aztreonam, and ciprofloxacin were more than 90% in multidrug-resistant *A. baumannii*. Carbapenems have been the choice in treating infections caused by *A. baumannii* [8]. However, the number of carbapenem-resistant *A. baumannii* strains has increased recently [19]. The acquisition of carbapenem resisrance in *A. baumannii* can be mainly due to the production of class D carbapenem hydrolyzing enzymes OXA-β-lactamases(OBLs)and class B metallo β-lactamases (MBLs) [20]. As shown in our study, sixty-four and 58 of the 64 multidrug-resistant strains studied here carried respectively *OXA-51* and *OXA-23* genes. Moreover, 58 isolates were positive for both *OXA-51* and *OXA-23* genes. our data support those of other studies demonstrated that *OXA-51* may be used as a marker to identify *A. baumannii* [21]. The *OXA-23* genes have been documented in strains associated with outbreaks of carbapenem resistant *A. baumannii* in Asia, Europe and South America [22]. Only one strain is *OXA-58* positive and all strains were negative for *OXA-24. OXA-58* belongs to *OXA-58* cluster, and *OXA-24* belongs to *OXA-40* cluster, which has been both reported in Europe and the United States.

And 103 of 120(85.8%) isolated are *VIM-1* positive. Our finding indicates that *VIM* -producing *A. baumannii* is more prevalent and that MBL-producing *A. baumannii* is increasing [5]. The percentage of wards with MBL-producing isolates might have been higher if a larger number of carbapenem-non susceptible isolates had been collected for this study.

Screening for genes encoding for AMEs demonstrated that 50 isolates contained the acetyltransferase gene *aac(6′)-Ib*. Few articles were published describing the presence of the aminoglycoside-encoding genes in strains isolated in china. In Guangzhou, Yang *et al.* have reported that 89.0% of 73 amikacin-resistant *A. baumannii*. were *aac(6′)-Ib* positive. And it is interesting that the *aac(6′)-Ib* gene is often present in integrons, transposons, and plasmids [23].

In addition to this enzymatic resistance, the loss of membrane permeability, due to alterations in specific porin, is an intrinsic carbapenem resistance mechanism in *A. baumannii*. A 29 kDa OMP, designated as CarO, if disrupted by insertion sequence, changes in the primary structure or decreased expression, would have a dramatic impact on the entry of antibiotic in the cell, thud contributing to resistance to this antibiotic [24]. But this is not limited to carbenem-resistant strains of *A. baumannii*. We showed the loss expression of *carO* in 77 strains. Further research on the outer membrane permeability is necessary.

Quaternary ammonium compounds(QAC) are used as antiseptics for the skin in hospitals. Until now many Gram-negative bacteria resistant to QAC have been reported. Resistance mechanisms are coded by *qacE* and *qac*Δ*E1*, whose products are transmembrane proteins [25]. The *qacE* and *qac E*Δ*1* genes are commonly found in Gram-negative bacteria, which are located in conserved sequences of integron class 1. The *qacE*Δ*1* gene is a mutation of the qacE gene, which acts as a multidrug transfer gene [26]. Sulfonamides act as a structural analogue of ρ-amino-benzoic acid and bind dihydropteroate synthase (DHPS), a catalytic enzyme in the folic acid biosynthesis pathway, resulting in the inhibition of dihydrofolic acid formation [27]. Resistance is conferred from the acquisition of an alternative DHPS gene (*sul*). One of the three known alternative DHPS genes, *sul1*, is usually located on the conserved region of integron class 1 [28]. The class 1 integrons are one of the most frequent elements in the acquisition, abundance, maintenance and spread of antimicrobial resistance gene cassettes among gram-negative bacteria isolated from clinical samples [29]. An integron possesses a gene for an integrase (*intI*), that permits the expression of gene cassettes incorporated in the variable region [30]. And 3′-conserved region at the end of the variable region contains the qacEΔ1 gene, followed by the sul1 gene. The class 1 integrons confer a benefit to the bacteria due to their ability to acquire gene cassettes that could provide advantages for survival in hostile environments [31]. We evaluated genetic elements, *sul1* and qacEΔ1 genes and genetic elements associated to lateral genetic transfer, intI1, genes, which comprise the genetic platforms of class 1 integrons. Genomic detection of *intI1* and *qac*Δ*E1-sul1* showed that 85 (70.8%) and 64 (53.3%) were positive, out of 120 *A. baumannii* isolates, respectively. Resistance genes related to QAC and sulfonamides antibiotics are both carried by class 1 integrons, so it increases concerns about gene expression that is resistant to QAC and sulfonamides, along with the increasing resistance to antibiotics by class 1 integrons. However, it is difficult to identify the sources of class 1 integrons from “environmental” or “clinical” one.

Pulsed-field gel electrophoresis (PFGE )which is based on the length polymorphism of bacterial chromosome DNA restriction fragments, is still considered the gold standard for typing outbreak-related isolates of *A. baumannii* [32]. It can be used to separate large digested DNA fragments, and then determine bacterial genotyping through the comparison of DNA band patterns. In many health care institutions, multidrug-resistant *A. baumannii* infection demonstrates complex epidemiologic profiles and coexistence of multiple strain type. PFGE results showed that among the multidrug-resistant *A. baumanii* strains in the hospital, DNA fingerprinting by pulsed-field gel electrophoresis showed fourteen clusters. Twenty-five of the 50 multidrug-resistant *A. baumannii* strains belonged to clone A, and type A1 was the most predominant (19 of 50 strains) in every ward. Based on the data presented, the interhospital transmission of multidrug-resistant *A. baumanii* isolates was apparent. These data also suggested that cross transmission between patients may contributed to the rise in the rates of multidrug resistance. Type A_2_, B, C_1_, C_2_, C_3_, C_4_ and other minor epidemic strains spread in different wards, which indicated that resistance towards antibiotic become more common in the hospital. In addition, type A_1_, B, C_3_ and C_4_ strains was found in ICU, type A_1_, A_2_, J and L in respiratory wards, type A_1_, A_2_, B, C_1_, C_2_, and G in neurology wards, and type A_2_, C_1_, D, and F in geriatrics ward, respectively, which suggested that the mix of multiple types of drug resistant strains make clinical treatment more difficult. Therefore, active surveillance is needed to detect and prevent the dissemination of such isolates.

The main limitation of this study is that it was confined to a single centre and it would be valuable to extend the origin of the strains. Another limitation is the small sample size which led to a lack of power to determine the individual effects of each broad spectrum antibiotics.

In conclusion, the emergence of broad-spectrum antibiotic resistance profiles in *A. baumannii* clinical isolates is worrying in the hospital. The mechanism of multidrug resistance in *A. baumannii* has not yet been fully understood. Multiple mechanisms are likely to work in synergism to produce this phenotype. our results highlight that enhanced surveillance and health policies for the detection and control of these MDR pathogens are urgently needed to avoid the emergence and spreading of such organism.
